# Converging crises and maternal and child health: colonialism, extreme weather, and COVID-19

**DOI:** 10.1186/s12978-025-02159-y

**Published:** 2025-11-05

**Authors:** Mislael Valentín-Cortés, Marie S. O’Neill, Carlos E. Rodríguez-Díaz, Michael R. Elliott, Paul J. Fleming, Alexis J. Handal

**Affiliations:** 1 University of Michigan School of Public Health, 1415 Washington Heights, Ann Arbor, MI 48109 USA; 2 Boston University School of Public Health, 715 Albany St, Boston, MA 02118 USA

**Keywords:** Maternal health, Child health, Disasters, Colonialism, Climate change, Puerto Rico, Health inequities, Reproductive health, COVID-19 pandemic, Environmental Justice

## Abstract

**Background:**

Climate change is a growing threat to human health, particularly in regions facing overlapping environmental hazards and social inequities. Puerto Rico—a U.S. territory with a colonial history—offers a unique case for examining how multiple disasters, including Hurricane Maria, ongoing earthquakes, and the COVID-19 pandemic, interact with structural vulnerabilities to affect maternal and child health. Despite increasing attention to climate-related health outcomes, little is known about the reproductive health impacts of cumulative disaster exposure in colonial contexts.

**Methods:**

We used U.S. National Vital Statistics System data (2017–2021) to assess associations between disaster exposure and six maternal and newborn outcomes: preterm birth, low birthweight, term low birthweight, gestational hypertension, gestational diabetes, and excessive weight gain. Disaster exposure was defined based on the timing of hurricanes and the pandemic, using a three-month lag period. We analyzed data from Puerto Rico and used Florida and Texas as comparison sites. Multivariable log-binomial regression models estimated adjusted prevalence ratios. Effect modification was tested for (1) region within Puerto Rico and (2) colonial status, comparing Puerto Rico (territory) to Florida and Texas (states). Simulations were conducted to account for potential live-birth bias.

**Results:**

Across 104,560 births in Puerto Rico, disaster periods were consistently associated with worse maternal health outcomes. For example, during the late post-hurricane period, gestational diabetes increased (RR = 1.19, 95% CI: 1.08, 1.31), while term low birthweight surprisingly appeared to decline (RR = 0.90, 95% CI: 0.83, 0.98). Associations with newborn health were mixed and may have been underestimated due to sharp declines in live births after disasters. Simulations suggested stronger disaster-related risks than observed in primary analyses. Effect modification by region and colonial status showed inconsistent but notable differences, particularly elevated maternal health risks in certain regions of Puerto Rico and compared to U.S. states.

**Conclusions:**

Our findings suggest that multiple disasters negatively affect reproductive health in Puerto Rico and that structural factors, including colonialism, may exacerbate these impacts. Public health responses must account for cumulative disaster exposure and systemic inequities to better support maternal and child health in marginalized settings, especially as climate change continues to intensify.

**Supplementary Information:**

The online version contains supplementary material available at 10.1186/s12978-025-02159-y.

## Abstract (plain language summary)

This study looks at how disasters and broader social conditions have affected the health of pregnant people and newborns in Puerto Rico. The U.S. territory experienced several major events—including a hurricane, a series of earthquakes, and the COVID-19 pandemic—that caused widespread disruption. These events coincided with long-standing inequalities in health, infrastructure, and emergency support compared to U.S. states. We analyzed over 100,000 birth records from Puerto Rico that occurred between 2017 and 2021 and compared them with similar data from Florida and Texas. We looked at health conditions during pregnancy such as gestational diabetes, excessive weight gain, early births, and low birthweight, and how these varied based on the timing of the birth relative to disasters. Our findings showed that disaster periods were associated with higher rates of gestational diabetes and high blood pressure, but that the effects on newborns were less clear, partly because fewer people gave birth during disaster periods. We also found some differences depending on the region of Puerto Rico and when we compared Puerto Rico to U.S. states, with people living in Puerto Rico (vs. Texas and Florida) and people in more vulnerable parts of the island (vs. the capital) were more likely to experience poor maternal health. Our results suggest that repeated disasters, combined with social and political factors like colonialism, can harm maternal health. Understanding these effects can help inform better policies and support systems for future emergencies—especially for communities that face structural disadvantages.

**Supplementary Information:**

The online version contains supplementary material available at 10.1186/s12978-025-02159-y.

## Background

Widespread disasters introduce stressors that disproportionately affect the physiological and psychological well-being of socially vulnerable populations, including racial and ethnic minorities, individuals experiencing poverty, women, and children [1–4]. Pregnant people and their newborns are particularly susceptible to these stressors due to the interplay of social, environmental, and biological vulnerabilities [5]. Studies have found that disasters such as hurricanes, floods, and earthquakes are associated with increased risks of preterm birth and low birthweight, with some reporting up to a 20–30% increase in preterm birth risk among exposed populations [6–9]. Moreover, disaster-related maternal stress has been linked to epigenetic changes affecting placental function and fetal development, suggesting pathways that extend beyond immediate birth outcomes to long-term child health impacts [8, 10]. Disasters can disrupt access to healthcare, interrupt prenatal services, cause trauma, and increase economic insecurity—factors known to heighten the risk of pregnancy complications [11–13]. Disaster-related food insecurity and healthcare disruptions can further endanger maternal and child health. Adequate nutrition during pregnancy is essential for fetal development, and disruptions in access to healthy food—often exacerbated by disaster-related supply chain disruptions or economic hardship—can result in adverse birth outcomes [14, 15]. Further, stress during pregnancy has been linked to increased production of oxytocin and inflammation, which can negatively affect gestational outcomes [16, 17]. In parallel, pregnant individuals experiencing depression or anxiety face a higher risk of preterm birth and other complications [18, 19]. When compounded by the precarious conditions introduced by disasters, these risks may be magnified and could result in post-natal and long-term consequences [20]. 

Epidemiologic literature on disasters and their effect on maternal and newborn health has focused on singular events, largely overlooking how cumulative and cascading disasters shape health outcomes. Studies on individual events, such as ice storms, hurricanes, and earthquakes, have linked disaster exposure to shortened gestation, reduced birthweight, and increased fetal loss [9, 21–23]. Disasters with profound impacts on health can be conceived of more broadly than just environmental hazards or pandemics. Other disruptive events with far-reaching implications for health include immigration raids and racially targeted policies, as they operate through similar psychosocial stress pathways. Such events can disproportionately impact historically marginalized communities, including affecting birth outcomes [24, 25] Despite growing evidence that climate change increases the frequency and severity of disasters, few epidemiologic studies have investigated how multiple disaster exposure affects maternal and child health, particularly in regions already affected by long-standing structural inequities. This is particularly concerning given evidence that recovery from one disaster is often incomplete before the next occurs, potentially leading to chronic stress, depleted social support systems, and worsening health inequities over time [26, 27]. 

Colonialism—particularly under U.S. imperialism—remains an underexamined driver of vulnerability in disaster and reproductive health research. Like racism and xenophobia, colonialism may operate as a structural determinant of health, shaping access to resources, infrastructure, and care [28]. Colonial systems result in economic extraction, disinvestment in public goods, and limit local autonomy—conditions that increase susceptibility to social and environmental hazards [29–32]. However, public health literature has largely treated colonialism as an external historical context, rather than a measurable determinant of health outcomes. Moreover, the intersection of colonialism with disaster vulnerability has received limited attention in reproductive health research, despite historical evidence that colonial systems exacerbate social, infrastructural, and health inequities during and after disasters [33, 34]. 

Puerto Rico offers a critical opportunity to examine how colonialism intersects with environmental disasters. The Puerto Rican archipelago remains a U.S. territory since it was acquired in 1898 and its residents lack full federal political representation and face structural disinvestment [35]. The territory’s ongoing economic crisis has been shaped by colonial policies, including the expiration of federal tax exemptions that triggered industrial collapse, austerity measures by an unelected fiscal control board, and a long-standing exclusion from equitable federal funding for Medicaid and disaster recovery [36]. These inequities have been especially visible in the aftermath of Hurricane Maria in 2017, where Puerto Rico received delayed and lower levels of federal aid compared to Texas and Florida after similar disasters [33, 34, 37]. Against this backdrop, Puerto Rico has faced multiple intersecting crises–Hurricane Maria, a series of earthquakes, and the COVID-19 pandemic—within a five year period. Understanding how these layered exposures affect reproductive health is essential to advance health equity and inform public health preparedness.

This study addresses two key gaps in the literature: the health impacts of multiple disaster exposure, and the role of colonialism as a modifier of reproductive health outcomes. Using national birth records from 2017 to 2021, we assess the association between disaster exposure and maternal and child health. We further examine whether the effects vary by geographic region in Puerto Rico or by colonial status, using Florida and Texas as non-colonial comparison sites. In doing so, this study contributes new evidence on how converging crises and structural inequities shape maternal and child health in politically marginalized settings. However, few studies have examined how multiple, cumulative disasters interact to shape maternal and child health outcomes, particularly in regions affected by structural inequities such as colonialism. Addressing these gaps is essential to understand and mitigate the disproportionate health burdens faced by marginalized populations.

## Methods

This study aimed to examine the association between multiple disaster exposure and maternal and child health outcomes in Puerto Rico, as well as the modifying role of geographic location and colonial status. We conducted a population-based, repeated cross-sectional analysis using data from the U.S. National Vital Statistics System (NVSS), maintained by the Centers for Disease Control and Prevention. These data include official records for all recorded Live births across U.S. states and territories. We obtained restricted-use birth files for the years 2017 to 2021 to incorporate geographic-level variables not available in public datasets.

Our analysis focused on births occurring in counties and municipalities in Puerto Rico, Texas, and Florida that were designated by the Federal Emergency Management Agency (FEMA) as eligible for individual-level disaster assistance. We excluded births from other jurisdictions to focus on areas with comparable disaster experiences. The University of Michigan Institutional Review Board deemed the study exempt from human subjects’ review (HUM00229614), since it relied solely on de-identified data.

Disaster exposure was classified based on the timing of births relative to the timing of disasters. To account for delayed and indirect disaster effects—including disrupted healthcare access, nutritional instability, and chronic stress—we applied a three-month lag from each disaster onset when determining exposure status. We chose a three-month lag period to balance considerations of both disaster impact timing and pregnancy physiology. From a disaster perspective, research indicates that the most severe disruptions to healthcare access, economic stability, and psychosocial wellbeing typically occur within the first two to three months after a disaster, representing a critical window of heightened community vulnerability [6, 34, 38, 39]. From a reproductive health perspective, we aimed to ensure that pregnancies spent at least one full trimester exposed to post-disaster conditions, since exposure timing is crucial for fetal development. For example, disaster exposure occurring only in the final weeks of pregnancy (e.g. at week 38) would have minimal influence on gestational duration or fetal growth. While a longer lag period (e.g., four or five months) could capture extended recovery challenges, it may dilute the acute disaster-specific impacts we sought to examine. Thus, a three-month lag maximized the likelihood of capturing both meaningful disaster exposure and biologically relevant pregnancy windows. We then categorized births into five mutually exclusive periods: pre-disaster (unexposed), early post-hurricane (0–6 months post-hurricane), late post-hurricane (6 + months post-hurricane to March 2020), early pandemic (March 12, 2020, to April 15, 2021), and late pandemic (April 25, 2021, onward, following broad vaccine availability in Puerto Rico). Earthquake exposure was not formally modeled due to lack of geographic precision and limited population-wide impact.

Six outcomes were drawn from birth records. For newborns, these included preterm birth (< 37 weeks), low birthweight (< 2,500 g), and term low birthweight (low birthweight among term infants). For maternal health, outcomes included physician-diagnosed gestational hypertension, gestational diabetes, and excessive weight gain. Preterm birth was defined using the obstetric estimate of gestational age, and excessive weight gain was classified according to CDC guidelines, which take into account pre-pregnancy body mass index (BMI) and total weight gained during pregnancy [40]. 

We calculated descriptive statistics for all variables and used multivariable log-binomial regression to estimate adjusted prevalence ratios (PRs) and 95% confidence intervals (Cis) for each outcome across disaster periods. The pre-disaster period served as the reference group in all models. Effect modification was tested using interaction terms for two factors: [1] region within Puerto Rico (based on the six regional divisions established by the Puerto Rico Tourism Company) and [2] colonial status. Covariates were selected based on epidemiologic principles of confounder adjustment, controlling for variables that were firstly, common causes of both disaster exposure and reproductive health outcomes, and, secondly, not on the causal pathway between exposure and outcome [41, 42]. All models were adjusted for maternal age, maternal education, marital status, pre-pregnancy BMI, paternal age, paternal education, enrollment in WIC, and birth payment method. For models assessing differences by colonial status (Puerto Rico vs. U.S. states), maternal race and a binary colonialism variable (Puerto Rico = colony; Texas and Florida = non-colony) were also included as covariates. These covariates were selected based on prior literature demonstrating their associations with both disaster vulnerability and maternal or child health outcomes [43, 44]. All statistical analyses were conducted using SAS OnDemand for Academics.

To address potential live-birth bias—whereby disaster-related reductions in births may result underestimation of adverse outcomes—we conducted supplemental simulations following Harville et al. and Liew et al. [45, 46] Using 2017 as the baseline year, we estimated the number of “missing births” from 2018 to 2021 and created hypothetical datasets by assigning elevated risks of adverse outcomes to these unobserved births. We then integrated simulated and observed data to estimate corrected risk ratios under varying assumptions, including 100% and 75% missingness scenarios. Full simulation results are provided in the supplementary materials.

## Results

Puerto Rico recorded 104,560 Live births between January 2017 and December 2021. A majority of birthing individuals had some level of college education (64.4%) and were between the ages of 15 and 34 (87.6%). Most were unmarried (69%) and over half (54.6%) had a pre-pregnancy BMI consistent with overweight or obese. High levels of financial need were evident, with over 84% participating in the Special Supplemental Nutrition Program for Woman, Infants, and Children (WIC), and 67.6% relying on Medicaid to cover birthing costs. Demographic characteristics of fathers showed similar trends. Full demographic information is presented in Table 1.

**Table 1:** Sociodemographic characteristics and distribution of key covariates among 104,560 Live births in Puerto rico; U.S. Vital statistics records, 2017–2021

	Pre-Disasters	Early Post-Hurricane	Late Post-Hurricane	Early pandemic	Late pandemic	*p*-value*
Births (n)	24,373	9.854	41,202	20,247	8.884	
Maternal Age						< 0.0001
< 15	0.1%	0.06%	0.06%	0.02%	0.02%	
15–24	43.7%	40.0%	40.0%	38.2%	36.8%	
25–34	45.1%	47.7.%	47.5%	49.0%	50.4%	
35–44	11.0%	12.2%	12.6%	12.6%	12.7%	
45–54	0.1%	0.1%	0.1%	0.1%	0.1%	
Maternal education						< 0.0001
High School or less	37.0%	35.6%	35.3%	34.4%	35.3%	
Some College	35.0%	33.0%	33.3%	33.5%	32.0%	
Bachelor’s or higher	28.0%	31.4%	31.4%	32.1%	32.7%	
Paternal Age						< 0.0001
< 15	0.0%	0.0%	0.0%	0.02%	0.0%	
15–24	28.6%	25.8%	25.9%	25.4%	24.5%	
25–34	49.0%	49.7%	49.5%	50.4%	51.1%	
35–44	19.2%	21.4%	21.1%	20.7%	20.9%	
45–54	3.1%	3.1%	3.5%	3.6%	3.4%	
Paternal education						< 0.0001
High School or less	47.6%	45.3%	45.8%	45.9%	46.2%	
Some College	35.1%	35.6%	34.7%	34.1%	34.0%	
Bachelor’s or higher	17.3%	19.1%	19.5%	20.0%	19.8%	
Payment Method						< 0.0001
Self-pay	1.6%	1.5%	2.0%	2.2%	2.3%	
Private insurance	28.7%	31.5%	30.7%	30.7%	30.4%	
Medicaid	69.5%	66.9%	67.2%	66.8%	67.1%	
Other	0.2%	0.1%	0.2%	0.3%	0.2%	
Marital status						0.0071
Single	69.4%	68.5%	69.4%	70.5%	69.5%	
Married	30.6%	31.5%	30.6%	29.5%	30.50%	
Body Mass Index						< 0.0001
Normal Weight	42.0%	41.2%	39.8%	38.1%	37.4%	
Underweight	6.3%	6.2%	5.8%	5.5%	5.0%	
Overweight	26.9%	27.8%	27.2%	27.7%	27.5%	
Obesity	20.7%	21.0%	22.5%	23.6%	24.7%	
Extreme Obesity	4.1%	3.8%	4.6%	5.1%	5.4%	
Region in PR						0.0038
Metro	28.2%	27.7%	28.5%	28.1%	27.7%	
Eastern	17.3%	17.9%	17.6%	18.3%	18.6%	
Central	12.2%	11.9%	11.9%	12.0%	11.4%	
Northern	13.0%	12.5%	13.0%	13.1%	12.3%	
Western	15.8%	16.5%	16.2%	15.9%	17.1%	
Southern	13.6%	13.6%	13.0%	12.6%	13.0%	

Disaster periods were associated with mixed effects on newborn health. Across most disaster periods, point estimates for low birthweight were significantly lower than in the pre-disaster periods. However, only the late post-hurricane period was associated with a statistically significant reduction in term low birthweight (PR = 0.90, 95% CI: 0.83, 0.98). No significant differences were observed for preterm birth across disaster periods (Table 2).

**Table 2:** Results from multivariable log-binomial regression models for reproductive outcomes in Puerto Rico by disaster periods (reference: pre-disaster); U.S. Vital records, 2017–2021

	Adjusted** prevalence ratios and 95% confidence intervals		
Disaster Period	Child Outcomes		
	Pre-term birth	Low birthweight	Term low birthweight
Early post-hurricane	1.01 (0.94, 1.09)	0.97 (0.90, 1.05)	0.90 (0.80, 1.02)
Late post-hurricane	1.01 (0.96, 1.07)	0.95 (0.90, 1.01)	0.90 (0.83, 0.98)*
Early pandemic	1.00 (0.94, 1.06)	0.98 (0.91, 1.05)	0.96 (0.87, 1.06)
Late pandemic	1.03 (0.95, 1.11)	1.03 (0.95, 1.12)	1.02 (0.91, 1.16)
	Maternal outcomes		
	Gestational hypertension	Gestational diabetes	Excessive weight gain
Early post-hurricane	1.05 (0.93, 1.19)	1.09 (0.95, 1.25)	1.01 (0.97, 1.05)
Late post-hurricane	1.19 (1.10, 1.29)*	1.19 (1.08, 1.31)*	1.07 (1.04, 1.10)*
Early pandemic	1.30 (1.18, 1.42)*	1.38 (1.24, 1.54)*	1.15 (1.11, 1.18)*
Late pandemic	1.21 (1.07, 1.36)*	1.37 (1.20, 1.56)*	1.13 (1.09, 1.17)*

*p-value < 0.05

**Models were adjusted for maternal age, maternal education, paternal age, paternal education, birth payment method, marital status, and pre-pregnancy body mass index

In contrast, maternal health outcomes showed more consistent patterns. Gestational hypertension, gestational diabetes, and excessive weight gain were generally elevated in all disaster periods, with the largest effects observed during the pandemic. For example, during the early pandemic, the prevalence of gestational diabetes increased by 38% (PR = 1.38, 95% CI: 1.24, 1.54), and gestational hypertension by 30% (PR = 1.30, 95% CI: 1.18, 1.42). Additional information is available in Table 2.

Geographic variation within Puerto Rico modified some associations. Compared to the Metropolitan region, newborns in the Central, Eastern, and Northern regions had consistently lower rates of term low birthweight during several disaster periods, although not all estimates reached statistical significance. For instance, during the early post-hurricane period, the Northern region showed a significantly lower risk of term low birthweight (PR = 0.62, 95% CI: 0.40, 0.98). In the late post-hurricane period, newborns in the Northern region continued to experience significantly lower rates (PR = 0.71, 95% CI: 0.54, 0.93) compared to those in the Metro area. Maternal health outcomes varied by region as well. In the Central region, individuals had elevated rates of gestational diabetes in both the early (PR = 1.48, 95% CI: 0.86, 2.54) and late (PR = 1.57, 95% CI: 1.07, 2.30) post-hurricane periods, though only the latter was statistically significant. During the early pandemic, gestational hypertension was significantly higher in the Eastern (PR = 1.40, 95% CI: 1.04, 1.84), Southern (PR = 1.57, 95% CI: 1.16, 2.10), and Western (PR = 1.27, 95% CI: 0.88, 1.68) regions. In the late pandemic period, gestational hypertension remained significantly elevated in the Southern region (PR = 1.72, 95% CI: 1.19, 2.49) compared to the Metropolitan region (Tables 3 and 4).

**Table 3:** Adjusted* prevalence ratios and 95% confidence intervals for associations between disaster periods and maternal and child health outcomes by region of Puerto rico; U.S. Vital records, 2017–2021

Region	Child health outcomes	Early Post Hurricane	Late Post Hurricane	Early Pandemic	Late Pandemic
Metro		reference	reference	reference	reference
Central	Pre-term birth	0.95 (0.72, 1.24)	1.01 (0.84, 1.21)	0.96 (0.77, 1.19)	1.13 (0.86, 1.50)
	Low birthweight	0.80 (0.60, 1.07)	0.93 (0.77, 1.13)	0.83 (0.66, 1.04)	0.89 (0.66, 1.19)
	Term low birthweight	0.65 (0.41, 1.01)	0.83 (0.62, 1.09)	0.80 (0.57, 1.13)	0.72 (0.46, 1.12)
Eastern	Pre-term birth	0.96 (0.76, 1.21)	0.96 (0.82, 1.12)	0.96 (0.80, 1.15)	1.15 (0.91, 1.46)
	Low birthweight	0.77 (0.60, 0.98)*	0.89 (0.75, 1.04)	0.87 (0.72, 1.05)	0.86 (0.67, 1.10)
	Term low birthweight	0.71 (0.49, 1.03)	0.92 (0.72, 1.18)	1.01 (0.76, 1.34)	0.83 (0.57, 1.19)
Northern	Pre-term birth	0.87 (0.67, 1.13)	0.96 (0.81, 1.14)	0.91 (0.74, 1.12)	1.04 (0.80, 1.36)
	Low birthweight	0.79 (0.59, 1.05)	0.89 (0.74, 1.08)	0.88 (0.70, 1.09)	0.82 (0.61, 1.09)
	Term low birthweight	0.62 (0.40, 0.98)*	0.74 (0.56, 0.99)	0.80 (0.57, 1.12)	0.66 (0.42, 1.04)
Southern	Pre-term birth	1.04 (0.81, 1.34)	0.98 (0.82, 1.16)	0.98 (0.79, 1.20)	1.03 (0.79, 1.35)
	Low birthweight	1.01 (0.79, 1.33)	0.83 (0.69, 1.00)	0.90 (0.72, 1.12)	0.92 (0.70, 1.22)
	Term low birthweight	1.08 (0.74, 1.57)	0.81 (0.61, 1.06)	1.01 (0.73, 1.39)	0.85 (0.57, 1.28)
Western	Pre-term birth	1.01 (0.79, 1.28)	1.05 (0.89, 1.24)	1.02 (0.84, 1.24)	1.13 (0.88, 1.46)
	Low birthweight	0.99 (0.77, 1.27)	0.96 (0.81, 1.14)	0.95 (0.78, 1.17)	0.98 (0.76, 1.27)
	Term low birthweight	0.95 (0.65, 1.38)	0.90 (0.70, 1.17)	1.06 (0.78, 1.43)	0.96 (0.66, 1.39)
	Maternal health outcomes				
Metro		reference	reference	reference	reference
Central	Gestational hypertension	0.68 (0.43, 1.08)	0.89 (0.67, 1.18)	0.84 (0.59, 1.18)	0.95 (0.61, 1.49)
	Gestational diabetes	1.48 (0.86, 2.54)	1.57 (1.07, 2.30)*	0.91 (0.58, 1.43)	1.16 (0.67, 2.03)
	Excessive weight gain	0.98 (0.85, 1.12)	0.98 (0.89, 1.07)	0.96 (0.86, 1.06)	1.00 (0.88, 1.14)
Eastern	Gestational hypertension	1.09 (0.76, 1.55)	1.09 (0.86, 1.39)	1.40 (1.06, 1.84)*	1.08 (0.75, 1.55)
	Gestational diabetes	0.83 (0.54, 1.26)	0.89 (0.68, 1.18)	0.89 (0.65, 1.22)	1.13 (0.77, 1.66)
	Excessive weight gain	0.98 (0.87, 1.11)	0.97 (0.90, 1.05)	0.97 (0.89, 1.07)	0.97 (0.87, 1.09)
Northern	Gestational hypertension	0.75 (0.46, 1.23)	0.80 (0.59, 1.10)	1.04 (0.73, 1.49)	0.80 (0.48, 1.31)
	Gestational diabetes	0.89 (0.52, 1.52)	0.66 (0.46, 0.95)	0.80 (0.53, 1.19)	0.91 (0.54, 1.51)
	Excessive weight gain	0.95 (0.83, 1.09)	0.96 (0.88, 1.04)	1.00 (0.91, 1.10)	1.00 (0.89, 1.13)
Southern	Gestational hypertension	1.06 (0.72, 1.56)	1.04 (0.80, 1.35)	1.57 (1.16, 2.10)*	1.72 (1.19, 2.49)*
	Gestational diabetes	1.13 (0.74, 1.71)	0.87 (0.65, 1.17)	0.92 (0.66, 1.28)	1.02 (0.67, 1.55)
	Excessive weight gain	0.98 (0.87, 1.12)	0.95 (0.87, 1.04)	0.95 (0.86, 1.05)	0.95 (0.83, 1.08)
Western	Gestational hypertension	1.04 (0.72, 1.49)	1.00 (0.79, 1.28)	1.27 (0.95, 1.68)	1.27 (0.88, 1.89)
	Gestational diabetes	0.77 (0.51, 1.16)	0.76 (0.58, 1.00)	0.92 (0.67, 1.24)	0.92 (0.63, 1.35)
	Excessive weight gain	1.00 (0.88, 1.13)	0.94 (0.86, 1.02)	0.95 (0.87, 1.05)	1.00 (0.90, 1.14)

*p-value < 0.05

** Models were adjusted for maternal age, maternal education, paternal age, paternal education, enrollment in WIC, birth payment method, marital status, and pre-pregnancy body mass index

**Table 4:** Sociodemographic characteristics and distribution of key covariates by colonialism status in florida, texas, and Puerto rico; births reported in U.S. Vital records, 2017–2021

	Texas and Florida	Puerto Rico	*p*-value
Maternal Age	*N* = 1,556,312	*N* = 104,560	< 0.0001
< 15	0.1%	0.1%	
15–24	23.5%	40.1%	
25–34	57.3%	47.5%	
35–44	18.9%	12.2%	
45–54	0.3%	0.1%	
WIC Use			< 0.0001
Uses WIC	40.2%	83.6%	
Payment Method			< 0.0001
Self-pay	5.5%	1.9%	
Private Insurance	43.1%	30.3%	
Medicaid	48.7%	67.6%	
Other	2.7%	0.2%	
Marital Status			< 0.0001
Single	45.4%	69.5%	
Married	54.6%	30.5%	
Maternal Education			< 0.0001
High school or less	42.2%	35.6%	
Some college	28.2%	33.6%	
Bachelor’s or higher	29.7%	30.8%	
Body Mass Index			< 0.0001
Normal Weight	40.9%	39.9%	
Underweight	3.3%	5.9%	
Overweight	27.8%	27.3%	
Obesity	23.0%	22.4%	
Extreme obesity	5.0%	4.6%	

Colonial status also modified some disaster-outcome associations. Newborn outcomes did not vary significantly between Puerto Rico and the U.S. states of Texas and Florida across any disaster periods. However, maternal outcomes differed. Puerto Rico exhibited consistently higher rates of gestational diabetes and excessive weight gain compared to the U.S. states. During the late pandemic period, gestational diabetes was significantly more common in Puerto Rico (PR = 1.17, 95% CI: 1.00, 1.31), as were excessive weight gain outcomes in the late post-hurricane (PR = 1.10, 95% CI: 1.07, 1.13), early pandemic (PR = 1.15, 95% CI: 1.12, 1.19), and late pandemic (PR = 1.20, 95% CI: 1.15, 1.24) periods (Table 5).

**Table 5:** Effect modification by colonialism for associations between maternal and newborn pregnancy outcomes and disaster periods showing results for Puerto Rico compared to the reference States (non-colony status) Florida and texas; U.S. Vital statistics records, 2017–2021

	Adjusted** prevalence ratios and 95% confidence intervals		
Disaster Period	Child Outcomes		
	Pre-term birth	Low birthweight	Term low birthweight
Early post-hurricane	1.00 (0.92, 1.08)	0.96 (0.89, 1.05)	0.92 (0.80, 1.05)
Late post-hurricane	0.97 (0.92, 1.03)	0.95 (0.90, 1.01)	0.92 (0.85, 1.01)
Early pandemic	0.94 (0.89, 1.01)	0.97 (0.91, 1.04)	0.97 (0.87, 1.08)
Late pandemic	0.96 (0.89, 1.04)	0.99 (0.91, 1.08)	1.00 (0.87, 1.14)
	Maternal outcomes		
	Gestational hypertension	Gestational diabetes	Excessive weight gain
Early post-hurricane	0.93 (0.82, 1.05)	1.05 (0.92, 1.21)	1.01 (0.97, 1.05)
Late post-hurricane	1.00 (0.92, 1.09)	1.06 (0.97 1.17)	1.10 (1.07, 1.13)*
Early pandemic	0.94 (0.86, 1.03)	1.06 (0.96, 1.18)	1.15 (1.12, 1.19)*
Late pandemic	0.91 (0.80, 1.03)	1.17 (1.00, 1.31)*	1.20 (1.15, 1.24)*

*p-value < 0.05

** Adjusted for maternal age, maternal education, maternal race, paternal age, paternal education, enrollment in WIC, birth payment method, marital status, and pre-pregnancy body mass index

To assess the impact of Live-birth bias, we conducted simulations assuming birth counts remained constant from 2017 to 2021. Births in Puerto Rico declined by 12–22% annually after Hurricane Maria, which may have led to underestimation of disaster-related health risks. Simulated scenarios suggested stronger associations than observed in the main models. For instance, corrected risk ratios for gestational diabetes reached up to 8.10 in 2021, while those for newborn health outcomes ranged from 0.92 to 2.86. These findings indicate that live-birth bias may have substantially suppressed the magnitude of observed associations in both the main effects and colonialism analyses. Full simulation results are presented in Table 6.

**Table 6:** Live-birth bias health supplemental analysis. Simulation of hypothetical risk scenarios assuming births stayed constant in PR; U.S. Vital statistics records, 2017–2021

Year	Risk of outcome among “missing” births	Corrected Relative Risk and 95% CI for PTB	Corrected Relative Risk and 95% CI for LBW	Corrected Relative Risk and 95% CI for GH	Corrected Relative Risk and 95% CI for GD
2018	100%	1.94 (1.85, 2.04)	1.99 (1.89, 2.09)	3.96 (3.66, 4.23)	5.06 (4.70, 5.44)
	50%	1.43 (1.36, 1.50)	1.43 (1.35, 1.50)	2.48 (2.30, 2.68)	3.08 (2.85, 3.33)
	20%	1.12 (1.06, 1.18)	1.09 (1.03, 1.15)	1.60 (1.48, 1.74)	1.90 (1.74, 2.06)
	18%	1.10 (1.04, 1.16)	1.07 (1.01, 1.13)	1.55 (1.42, 1.68)	1.82 (1.67, 1.98)
	15%	1.07 (1.01, 1.13)	1.03 (0.97, 1.09)	1.46 (1.34, 1.59)	1.70 (1.56, 1.85)
	12%	1.04 (0.98, 1.10)	1.00 (0.94, 1.06)	1.37 (1.26, 1.49)	1.58 (1.45, 1.72)
	10%		0.98 (0.92, 1.03)	1.31 (1.21, 1.43)	1.50 (1.37, 1.64)
	8%			1.25 (1.15, 1.37)	1.42 (1.30, 1.56)
	5%			1.05 (0.96, 1.15)	1.31 (1.19, 1.43)
	3%			1.04 (0.95, 1.13)	1.23 (1.12, 1.35)
2019	100%	2.27 (2.16, 2.38)	2.34 (2.23, 2.46)	4.97 (4.63, 5.34)	6.40 (5.96, 6.87)
	50%	1.56 (1.48, 1.65)	1.57 (1.49, 1.66)	2.98 (2.76, 3.21)	3.70 (3.43, 3.99)
	20%	1.14 (1.08, 1.20)	1.11 (1.05, 1.17)	1.78 (1.65, 1.93)	2.08 (1.92, 2.26)
	18%	1.11 (1.05, 1.17)	1.08 (1.02, 1.14)	1.70 (1.57, 1.85)	1.98 (1.82, 2.15)
	15%	1.07 (1.01, 1.13)	1.03 (0.97, 1.09)	1.58 (1.46, 1.72)	1.81 (1.67, 1.97)
	12%	1.03 (0.97, 1.08)	0.99 (0.93, 1.04)	1.46 (1.35, 1.59)	1.65 (1.52, 1.80)
	10%		0.96 (0.90, 1.01)	1.38 (1.27, 1.51)	1.54 (1.41, 1.69)
	8%			1.30 (1.20, 1.42)	1.44 (1.31, 1.57)
	5%			1.18 (1.09, 1.29)	1.28 (1.19, 1.37)
	3%			1.11 (1.01, 1.21)	1.17 (1.06, 1.28)
2020	100%	2.72 (2.59, 2.85)	2.86 (2.72, 3.00)	6.56 (6.12, 7.03)	8.57 (7.99, 9.20)
	50%	1.75 (1.66, 1.84)	1.80 (1.71, 1.90	3.79 (3.53, 4.08)	4.83 (4.49, 5.20)
	20%	1.17 (1.11, 1.24)	1.17 (1.11, 1.24)	2.14 (1.98, 2.31)	2.58 (2.39, 2.80)
	18%	1.13 (1.07, 1.20)	1.13 (1.07, 1.19)	2.02 (1.87, 2.19)	2.43 (2.25, 2.64)
	15%	1.07 (1.02, 1.13)	1.07 (1.01, 1.13)	1.86 (1.72, 2.01)	2.21 (2.04, 2.40)
	12%	1.02 (0.96, 1.07)	1.00 (0.95, 1.06)	1.69 (1.56, 1.84)	1.98 (1.83, 2.16)
	10%		0.96 (0.91, 1.02)	1.58 (1.46, 1.72)	1.83 (1.69, 2.00)
	8%			1.47 (1.35, 1.60)	1.69 (1.55, 1.84)
	5%			1.31 (1.20, 1.42)	1.46 (1.34, 1.60)
	3%			1.20 (1.10, 1.31)	1.31 (1.20, 1.44)
2021	100%	2.63 (2.51, 2.76)	2.75 (2.62, 2.89)	6.16 (5.75, 6.61)	8.10 (7.55, 8.69)
	50%	1.73 (1.64, 1.82)	1.77 (1.68, 1.87)	3.61 (3.36, 3.88)	4.64 (4.31, 5.00)
	20%	1.19 (1.13, 1.26)	1.18 (1.12, 1.25)	2.08 (1.92, 2.25)	2.57 (2.37, 2.78)
	18%	1.15 (1.09, 1.22)	1.14 (1.08, 1.21)	1.98 (1.83, 2.14)	2.43 (2.24, 2.63)
	15%	1.10 (1.04, 1.16)	1.09 (1.03, 1.15)	1.82 (1.68, 1.97)	2.22 (2.05, 2.41)
	12%	1.05 (0.99, 1.11)	1.03 (0.97, 1.09)	1.67 (1.54, 1.81)	2.01 (1.85, 2.19)
	10%		0.99 (0.93, 1.05)	1.57 (1.44, 1.70)	1.87 (1.72, 2.04)
	8%			1.46 (1.35, 1.59)	1.74 (1.59, 1.89)
	5%			1.31 (1.20, 1.43)	1.53 (1.40, 1.67)
	3%			1.21 (1.11, 1.32)	1.39 (1.27, 1.52)

* 2017 (pre-disaster) is used as the reference point for all comparisons

** Starting point for simulation “risk” was based on baseline (i.e., 2017) risk for outcome. PTB was 11.5%, LBW was 10.0%, GH was 4%, and GD was 3%

## Discussion

This study examined the association between multiple disaster exposures and maternal and child health among 104,560 live births recorded in Puerto Rico’s vital records from 2017 to 2021. It also assessed effect modification by [1] geographic location within Puerto Rico and [2] colonialism, by comparing associations in Puerto Rico to those in Texas and Florida.

First, disaster periods were consistently associated with adverse maternal health outcomes, with particularly strong effects observed during the early and late pandemic periods. Second, disaster exposure did not significantly impact child health in Puerto Rico, and these associations did not vary by colonialism or geographic location. Third, although geographic-level effect modification results were mixed, gestational diabetes rates were higher in the early and late post-hurricane periods in Central and Eastern Puerto Rico compared to the Metropolitan region. Additionally, gestational hypertension rates were higher in the early and late pandemic periods in Southern Puerto Rico compared to the Metropolitan region. These findings are particularly notable given that Central and Eastern Puerto Rico were anecdotally among the most affected by Hurricane Maria, while the Southern region was heavily impacted by the earthquake events that coincided with the early pandemic. Lastly, colonialism modified the effects of disaster exposure on gestational diabetes in the late pandemic period and on excessive weight gain in the late post-hurricane, early pandemic, and late pandemic periods, with Puerto Rico experiencing a greater burden than Texas and Florida.

Overall, our findings share similarities with existing research while also highlighting key differences. While disaster exposure was associated with adverse maternal health outcomes, these associations remain underexplored in prior studies, and it is unclear whether our findings would be replicable in other populations. Nonetheless, our results suggest that pregnant individuals in Puerto Rico experienced heightened rates of post-disaster gestational hypertension, gestational diabetes, and excessive weight gain, with the strongest associations observed during the early pandemic period. This surge may reflect the effects of the pandemic alone or the cumulative impact of prior disasters compounded by the pandemic. However, we are unable to determine the relative contribution of these factors.

Prior studies have linked the COVID-19 pandemic to increased pregnancy-related stress and anxiety, driven by factors such as social isolation, unemployment, poverty, and intimate partner violence [47–49]. These psychosocial stressors, combined with heightened risks of food insecurity and disruptions to food systems during the pandemic, may have contributed to the elevated rates of adverse maternal health outcomes observed in our study [50, 51]. Additionally, these associations may reflect the cumulative effects of multiple disasters. Hurricane Maria, for example, was similarly associated with adverse mental health outcomes and disruptions in food systems in Puerto Rico [52–57]. 

Notably, our analysis of newborn health outcomes (Table 2) does not align with our initial hypothesis and shows some inconsistencies with prior studies. While our study did not find significant post-disaster differences in preterm birth or low birthweight, previous research has reported adverse impacts on children born in post-disaster settings. In particular, studies examining the effects of hurricanes, wildfires, and floods have found increased rates of preterm birth and low birthweight among disaster-exposed individuals, particularly among racial and ethnic minorities [7, 58–61]. However, prior research suggests that such findings may be influenced by live-birth bias. A key example is Hamilton and colleagues, who analyzed official birth records following Hurricane Katrina and initially found apparent reductions in preterm birth and low birthweight after the storm [62]. These findings were later challenged by researchers who estimated hypothetical birth rates under stable conditions and found that Hurricane Katrina was Likely associated with worse outcomes, with estimated risk ratios ranging from 1.30 to 1.35—contradicting the initial results [45, 62, 63]. Similarly, we found evidence of Live-birth bias in our analysis, given the significant decline in yearly Live births in Puerto Rico following Hurricane Maria. In 2017, there were 24,373 births, but this number steadily declined to 19,332 in 2021, representing a 21% reduction. More information can be seen in Table 7. It is likely that stable birth rates would have yielded different results in our analysis. However, this decline in total live births is plausibly linked, at least in part, to the impact of multiple disasters. Further research is needed to determine whether these reductions were driven by declining fertility, increased infant mortality, migration, or other factors affecting childbearing.

**Table 7:** Total births by year and percent reduction in total Live births in florida, texas, and Puerto rico; U.S. Vital statistics records, 2017–2021

Year	Puerto Rico	Texas	Florida
2017	24,373 (baseline)	115,449 (baseline)	206,031 (baseline)
2018	21,482 (12% reduction)	114,336 (1% reduction)	204,991 (1% reduction)
2019	20,408 (16% reduction)	113,226 (2% reduction)	203,259 (1% reduction)
2020	18,965 (22% reduction)	109,301 (5% reduction)	193,538 (6% reduction)
2021	19,332 (21% reduction)	109,760 (5% reduction)	199,602 (3% reduction)
Total	104,560	562,072	1,007,421

To the best of our knowledge, this study is among the few epidemiologic investigations to examine the impact of multiple disasters while considering colonialism as a determinant of health. While our results were mixed, the direction of association for gestational diabetes and excessive weight gain was consistently harmful. Stronger associations in Puerto Rico compared to Texas and Florida suggest that colonialism may have heightened the risk of disaster-related adverse maternal health outcomes. Additionally, live-birth bias, as discussed earlier, may have led to an underestimation of colonialism’s effects on maternal and child health. Puerto Rico experienced a substantial yearly decline in total Live births, ranging from 12 to 22%, whereas Texas and Florida saw only a 1–6% reduction (Table 6). This stark disparity suggests that colonialism may contribute to a differential reduction in live births under multiple disaster scenarios, further exacerbating health inequities.

Overall, geographic location within Puerto Rico did not significantly modify the relationship between disaster exposure and maternal and child health. However, some effect estimates in Table 3 suggest that proximity to disaster epicenters and more severe disaster experiences may be associated with adverse maternal health outcomes. Specifically, gestational diabetes was higher among individuals in the Central region during both the early (PR = 1.48, 95% CI: 0.86, 2.54) and late post-hurricane periods (PR = 1.57, 95% CI: 1.07, 2.30) compared to those in the Metropolitan region. Additionally, gestational hypertension was elevated in the Southern region during both the early (PR = 1.57, 95% CI: 1.16, 2.10) and late pandemic periods (PR = 1.72, 95% CI: 1.19, 2.49) compared to the Metropolitan region. These findings are particularly notable, as the Central region was reported to have experienced significant hurricane-related complications due to resource scarcity, while the Southern region was the epicenter of the earthquakes that occurred during the early pandemic period [64, 65]. These results align with prior research, including a study in China that found stronger adverse mental health effects among pregnant individuals living closer to earthquake epicenters [66]. 

### Strengths & limitations

This study has several strengths. Importantly, this study uses a novel approach to examining colonialism as a determinant of health in the context of multiple disaster exposures—an area that remains largely understudied. Our observational and exploratory analysis provides an initial foundation for understanding these associations and guiding future research. Additionally, while cross-sectional designs traditionally limit causal inference, our approach mitigates some of these challenges by leveraging precise birth dates from official records, allowing us to compare outcomes against disaster timelines and reduce the risk of temporal ambiguity and reverse causation.

The study also benefits from the comprehensive nature of the NVSS, which serves as the official record of all live births in the U.S. This ensures that our dataset is fully representative of the study population. Furthermore, the use of geographic-level zip code data combined with FEMA disaster declarations allowed us to focus on areas most affected by disasters, reducing the risk of exposure misclassification. Lastly, the reliance on clinically diagnosed maternal health outcomes—certified and recorded by physicians—minimizes the potential for outcome misclassification, strengthening the validity of our findings.

Our study has several limitations to consider. First, the vital records data lacked unique identifiers, preventing us from accounting for multiple births from the same individuals. However, given that most births occur to first-time or non-consecutive repeat mothers, the likelihood of multiple births from the same individuals within the study period is relatively low. As a result, while this may introduce minor clustering effects, it is unlikely to significantly impact the overall observed associations. Second, selection bias may have influenced our findings, as the documented population exodus from Puerto Rico following disasters could include individuals at higher or lower risk for adverse outcomes, potentially underestimating or overestimating the true impact. Additionally, pregnancies most affected by disasters may have been more likely to result in miscarriage, stillbirth, or abortion, leading to an underestimation of observed associations [67]. 

While our study attempted to adjust for key confounders available in the data, residual confounding cannot be ruled out, as vital records do not capture key social and behavioral factors such as income, employment, or food security—factors that could influence pregnancy outcomes and be linked to disaster exposure. Moreover, our exposure classification does not account for individual-level variability in disaster experiences, which may bias our results toward the null.

The study lacks adjustments for multiple comparisons, increasing the possibility that some observed associations occurred by chance. We opted not to apply a Bonferroni correction, as it may be overly conservative and increase the risk of type II errors [68, 69]. Future studies may consider using false discovery rate (FDR) adjustments, which better balance the risk of type I and type II errors in analyses with multiple hypotheses [70]. 

Our live-birth bias analysis must be interpreted within the context of Puerto Rico’s long-term demographic trends. The island was already experiencing a significant decline in birth rates due to an ongoing economic crisis, which Likely contributed to emigration and slowed population growth. This trend may be attributed to the ongoing economic crisis that led to emigration and reduced population growth. Prior to Hurricane Maria in September 2017, Puerto Rico was already experiencing a significant population decline due to long-term economic challenges [71, 72]. However, Hurricane Maria in 2017 exacerbated this decline, leading to the steepest year-over-year drop in live births—from 28,000 in 2016 to 24,000 in 2017 and 21,000 in 2018. While birth rates began stabilizing between 2020 and 2023, this stabilization may have occurred sooner if not for the compounded effects of multiple disasters. These trends highlight how existing demographic and economic challenges intensified following the disasters, shaping Puerto Rico’s population trajectory. Nonetheless, we are unable to fully disentangle the effects of the preexisting economic crisis from the disaster aftermath when accounting for live-birth bias.

Further complicating our analysis is the issue of colonialism. Notably, Puerto Rico has long faced elevated rates of PTB and LBW, with studies showing that Puerto Rican women have a preterm birth rate of 13.2%, higher than other U.S. Hispanic subgroups [73]. Prior to Hurricane María, Puerto Ricans had a 23% higher rate of preterm birth, and a 35% higher rate of low birth weight compared to pregnancies in the continental United States [74]. These elevated rates are likely driven by systemic issues rooted in colonialism, including underfunded healthcare infrastructure, economic instability, and social inequities. Given this historically high baseline, the relative impact of these disasters on birth outcomes may appear less pronounced than expected. Moreover, factors such as declining birth rates during the preceding economic crisis and post-disaster population shifts may lead to misleading findings if the full context is not considered. Further, in our study, we operationalized colonialism as residing in a U.S. colony versus not residing in one. However, colonialism is a complex system shaped by numerous individual, community, political, and economic factors—including generational trauma, structural inequities, racism, and indigenous identity. These intersecting influences likely contribute to how colonialism affects maternal and child health outcomes. The lack of individual-level data in vital records limited our ability to explore these pathways. However, qualitative research based on in-depth interviews (currently in preparation) may provide additional insights that complement this analysis. Additionally, while birth vital records are a useful population-based data source, their design prioritizes de-identification in order to protect individual privacy, limiting the availability of detailed socioeconomic and geographic variables. However, our analysis adjusted for multiple SES proxies, including maternal and paternal education, maternal race, maternal and paternal age, marital status, birth payment method, and WIC participation. While direct income data was unavailable, these covariates capture key dimensions of SES, as WIC participation and Medicaid coverage during birth both require low-income eligibility. Therefore, while some residual confounding remains possible, it is likely minimal. We also considered alternative modeling approaches such as spatial, instrumental variable, and Bayesian hierarchical models to address unmeasured confounding; however, spatial models were not appropriate given our time-based exposure, instrumental variables were infeasible, and hierarchical models were limited by small sample sizes in some regions and concentration in the Metropolitan area. Future research linking vital records with richer data sources may further strengthen SES measurement and adjustment.

## Conclusion

Overall, our findings provide important insights into the relationship between multiple disaster exposures and maternal and child health. As hypothesized, gestational hypertension, gestational diabetes, and excessive weight gain were adversely impacted in post-disaster periods. Additionally, both geographic region within Puerto Rico and colonialism modified some of the associations between disaster exposure and maternal health. Our main effects analysis did not find significant associations between disaster exposure and preterm birth, low birthweight, or term low birthweight. However, these results may not fully capture the true impact due to potential live-birth bias and the substantial decline in yearly live births following disasters. Beyond Puerto Rico, these findings have broader implications for disaster preparedness, reproductive health, and climate resilience in other historically marginalized and structurally disadvantaged populations. Understanding how multiple disasters, particularly within the context of colonialism, impact maternal and child health is critical for informing public health strategies, emergency response planning, and long-term policy interventions. As climate change intensifies the frequency and severity of disasters, these insights can help shape policies that prioritize equitable disaster response, strengthen maternal health services in post-disaster settings, and mitigate the disproportionate burden on vulnerable populations.

These findings have direct implications for public policy and disaster preparedness planning. For example, the observed increases in gestational diabetes, hypertension, and excessive weight gain highlight the need for targeted maternal health interventions in post-disaster settings, such as ensuring uninterrupted prenatal care, expanding nutritional support programs, and integrating mental health services into emergency response frameworks. Additionally, recognizing the modifying role of colonialism underscores the importance of equitable federal aid allocation and strengthening local governance capacities to reduce structural barriers to health. Policymakers should prioritize developing disaster response strategies that address both immediate healthcare needs and longer-term social determinants of health to mitigate the disproportionate impacts on pregnant people and newborns in marginalized communities.

Collectively, our findings highlight the urgency of addressing systemic inequities in disaster response and recovery. Future research could benefit from refined exposure assessments and retrospective designs that more carefully account for individual disaster experiences, levels of exposure, and additional maternal and child health outcomes across multiple disaster events. By integrating these perspectives into public health and policy frameworks, we can better support maternal and child health resilience in disaster-prone and politically marginalized regions worldwide.

## Supplementary Information

Supplementary Material 1.

## Data Availability

The datasets generated and/or analyzed during the current study are not publicly available due to data use agreements with the National Center for Health Statistics. Access to restricted-use vital statistics data can be requested through the CDC Research Data Center (https://www.cdc.gov/rdc/).

## Abbreviations

BMI: Body Mass Index
CDC: Centers for Disease Control and Prevention
CI: Confidence Interval
FEMA: Federal Emergency Management Agency
FDR: False Discovery Rate
LBW: Low Birthweight
NVSS: National Vital Statistics System
PR: Prevalence Ratio
PTB: Preterm Birth
U.S.: United States
WIC: Special Supplemental Nutrition Program for Women, Infants, and Children
