# Supplementary material for: Converging crises and maternal and child health: colonialism, extreme weather, and COVID-19
Source: Reprod Health. 2025 Nov 5;22:222. doi: 10.1186/s12978-025-02159-y (PMC12590636; doi:10.1186/s12978-025-02159-y)
Supplement: Supplementary file 1 — Supplementary Material 1. [file 12978_2025_2159_MOESM1_ESM.docx]

**Supplemental table 1:** Live-birth bias health supplemental analysis. Simulation of hypothetical risk scenarios assuming births stayed constant (75%) in PR; U.S. Vital Statistics Records, 2017-2021.

| **Year** | **Risk of outcome among “missing” births** | **Corrected Relative Risk and 95% CI for PTB** | **Corrected Relative Risk and 95% CI for LBW** | **Corrected Relative Risk and 95% CI for GH** | **Corrected Relative Risk and 95% CI for GD** |
| --- | --- | --- | --- | --- | --- |
| **2018** | 100% | 1.69 (1.60, 1.77) | 1.71 (1.62, 1.80) | 3.21 (2.98, 3.45) | 4.07 (3.78, 4.38) |
|  | 50% | 1.30 (1.23, 1.37) | 1.29 (1.22, 1.36) | 2.11 (1.96, 2.29) | 2.59 (2.39, 2.80) |
|  | 20% | 1.07 (1.01, 1.13) | 1.03 (0.97, 1.09) | 1.46 (1.34, 1.59) | 1.70 (1.56, 1.85) |
|  | 18% | 1.05 (1.00, 1.11) | 1.02 (0.96, 1.08) | 1.41 (1.30, 1.54) | 1.64 (1.50, 1.79) |
|  | 15% | 1.03 (0.97, 1.09) | 0.99 (0.93, 1.05) | 1.35 (1.24, 1.47) | 1.55 (1.42, 1.69) |
|  | 12% | 1.01 (0.95, 1.06) | 0.96 (0.91, 1.02) | 1.28 (1.18, 1.40) | 1.46 (1.34, 1.60) |
|  | 10% |  | 0.95 (0.89, 1.00) | 1.24 (1.14, 1.35) | 1.40 (1.28, 1.53) |
|  | 8% |  |  | 1.20 (1.10, 1.30) | 1.34 (1.23, 1.47) |
|  | 5% |  |  | 1.03 (0.95, 1.13) | 1.26 (1.15, 1.38) |
|  | 3% |  |  | 1.03 (0.94, 1.13 | 1.20 (1.09, 1.31) |
| **2019** | 100% | 1.92 (1.82, 2.01) | 1.96 (1.86, 2.06) | 3.97 (3.70, 4.27) | 5.05 (4.70, 5.43) |
|  | 50% | 1.39 (1.32, 1.46) | 1.38 (1.31, 1.46) | 2.48 (2.30, 2.68) | 3.03 (2.80, 3.27) |
|  | 20% | 1.07 (1.01, 1.13) | 1.03 (0.97, 1.09) | 1.58 (1.46, 1.72) | 1.81 (1.67, 1.97) |
|  | 18% | 1.05 (0.99, 1.11) | 1.01 (0.95, 1.07) | 1.52 (1.40, 1.65) | 1.73 (1.59, 1.89) |
|  | 15% | 1.02 (0.96, 1.07) | 0.97 (0.92, 1.03) | 1.43 (1.32, 1.56) | 1.61 (1.48, 1.76) |
|  | 12% | 0.98 (0.93, 1.04) | 0.94 (0.89, 1.00) | 1.34 (1.24, 1.46( | 1.49 (1.36, 1.63) |
|  | 10% |  | 0.92 (0.86, 0.97) | 1.28 (1.18, 1.40) | 1.41 (1.29, 1.54) |
|  | 8% |  |  | 1.22 (1.12, 1.33) | 1.33 (1.21, 1.45) |
|  | 5% |  |  | 1.14 (1.04, 1.24) | 1.21 (1.13, 1.30) |
|  | 3% |  |  | 1.08 (0.98, 1.17) | 1.13 (1.03, 1.24) |
| **2020** | 100% | 2.23 (2.13, 2.34) | 2.33 (2.22, 2.45) | 5.18 (4.82, 5.56) | 6.70 (6.24, 7.20) |
|  | 50% | 1.51 (1.43, 1.59) | 1.54 (1.46, 1.62) | 3.10 (2.88, 3.34) | 3.89 (3.61, 4.20) |
|  | 20% | 1.07 (1.02, 1.13) | 1.07 (1.01, 1.13) | 1.86 (1.72, 2.01) | 2.21 (2.04, 2.40) |
|  | 18% | 1.05 (0.99, 1.10) | 1.03 (0.98, 1.09) | 1.78 (1.64, 1.92) | 2.10 (1.93, 2.28) |
|  | 15% | 1.00 (0.95, 1.06) | 0.99 (0.93, 1.05) | 1.65 (1.52, 1.79) | 1.93 (1.77, 2.10) |
|  | 12% | 0.96 (0.91, 1.01) | 0.94 (0.89, 1.00) | 1.53 (1.41, 1.66) | 1.76 (1.62, 1.92) |
|  | 10% |  | 0.91 (0.86, 0.96) | 1.44 (1.33, 1.57) | 1.65 (1.51, 1.80) |
|  | 8% |  |  | 1.36 (1.25, 1.48) | 1.54 (1.41, 1.68) |
|  | 5% |  |  | 1.24 (1.14, 1.35) | 1.37 (1.25, 1.50) |
|  | 3% |  |  | 1.16 (1.06, 1.26) | 1.25 (1.14, 1.38) |
| **2021** | 100% | 2.18 (2.07, 2.29) | 2.26 (2.15, 2.38) | 4.89 (4.55, 5.25) | 6.37 (5.93, 6.84) |
|  | 50% | 1.50 (1.43, 1.58) | 1.53 (1.45, 1.61) | 2.97 (2.76, 3.20) | 3.78 (3.50, 4.07) |
|  | 20% | 1.10 (1.04, 1.16) | 1.09 (1.03, 1.15) | 1.82 (1.68, 1.97) | 2.22 (2.05, 2.41) |
|  | 18% | 1.07 (1.02, 1.13) | 1.06 (1.00, 1.12) | 1.75 (1.61, 1.89) | 2.12 (1.95, 2.30) |
|  | 15% | 1.03 (0.98, 1.09) | 1.01 (0.95, 1.07) | 1.63 (1.50, 1.77) | 1.96 (1.80, 2.13) |
|  | 12% | 0.99 (0.94, 1.05) | 0.97 (0.91, 1.03) | 1.52 (1.40, 1.65) | 1.81 (1.66, 1.96) |
|  | 10% |  | 0.94 (0.88, 0.99) | 1.44 (1.32, 1.56) | 1.70 (1.56, 1.85) |
|  | 8% |  |  | 1.36 (1.25, 1.48) | 1.60 (1.46, 1.74) |
|  | 5% |  |  | 1.25 (1.14, 1.36) | 1.44 (1.32, 1.58) |
|  | 3% |  |  | 1.17 (1.07, 1.28) | 1.34 (1.22, 1.46) |

* 2017 (pre-disaster) is used as the reference point for all comparisons

** PTB = Preterm birth, LBW = Low birthweight, GH = Gestational Hypertension, GD = Gestational Diabetes

*** Starting point for simulation “risk” was based on baseline (i.e., 2017) risk for outcome. PTB was 11.5%, LBW was 10.0%, GH was 4%, and GD was 3%.

**Supplemental Table 2:** Live-birth bias colonialism supplemental analysis. Simulation of hypothetical risk scenarios assuming births stayed constant in PR; U.S. Vital Statistics Records, 2017-2021.

| **Year** | **Risk of outcome among “missing” births** | **Corrected Relative Risk and 95% CI for PTB** | **Corrected Relative Risk and 95% CI for LBW** | **Corrected Relative Risk and 95% CI for GH** | **Corrected Relative Risk and 95% CI for GD** |
| --- | --- | --- | --- | --- | --- |
| **2018** | 100% | 2.42 (2.39, 2.45) | 2.76 (2.72, 2.79) | 2.57 (2.53, 2.60) | 2.62 (2.59, 2.66) |
|  | 50% | 1.66 (1.62, 1.69) | 1.77 (1.73, 1.81) | 1.45 (1.40, 1.49) | 1.58 (1.54, 1.63) |
|  | 20% | 1.25 (1.21, 1.29) | 1.30 (1.26, 1.34) | 0.93 (0.87, 0.98) | 0.97 (0.92, 1.03) |
|  | 18% | 1.22 (1.18, 1.26) | 1.27 (1.26, 1.34) | 0.89 (0.84, 0.95) | 0.93 (0.87, 0.99) |
|  | 15% | 1.18 (1.14, 1.22) | 1.22 (1.18, 1.26) | 0.84 (0.79, 0.90) | 0.87 (0.81, 0.93) |
|  | 12% | 1.14 (1.10, 1.18) | 1.18 (1.13, 1.22) | 0.79 (0.73, 0.85) | 0.81 (0.75, 0.87) |
|  | 10% |  | 1.15 (1.10, 1.19) | 0.76 (0.70, 0.81) | 0.77 (0.71, 0.83) |
|  | 8% |  |  | 0.72 (0.66, 0.78) | 0.73 (0.66, 0.79) |
|  | 5% |  |  | 0.60 (0.54, 0.66) | 0.67 (0.60, 0.74) |
|  | 3% |  |  | 0.60 (0.53, 0.66) | 0.63 (0.56, 0.70) |
| **2019** | 100% | 2.87 (2.84, 2.90) | 3.44 (3.41, 3.47) | 2.68 (2.65, 2.72) | 2.93 (2.90, 2.97) |
|  | 50% | 1.80 (1.76. 1.83) | 2.10 (2.06, 2.14) | 1.62 (1.58, 1.66) | 1.73 (1.69, 1.78) |
|  | 20% | 1.24 (1.20, 1.28) | 1.41 (1.37, 1.45) | 0.98 (0.93, 1.03) | 1.00 (0.94, 1.05) |
|  | 18% | 1.21 (1.17, 1.25) | 1.36 (1.32, 1.40) | 0.94 (0.89, 0.99) | 0.95 (0.89, 1.00) |
|  | 15% | 1.16 (1.12, 1.20) | 1.30 (1.26, 1.34) | 0.87 (0.82, 0.93) | 0.87 (0.82, 0.93) |
|  | 12% | 1.10 (1.06, 1.14) | 1.23 (1.19, 1.28) | 0.81 (0.75, 0.86) | 0.80 (0.74, 0.86) |
|  | 10% |  | 1.19 (1.15, 1.23) | 0.77 (0.71, 0.82) | 0.75 (0.68, 0.81) |
|  | 8% |  |  | 0.72 (0.66, 0.78) | 0.70 (0.63, 0.76) |
|  | 5% |  |  | 0.66 (0.60, 0.72) | 0.62 (0.55, 0.69 |
|  | 3% |  |  | 0.61 (0.55, 0.68) | 0.57 (0.50, 0.64) |
| **2020** | 100% | 3.77 (3.74, 3.80) | 4.57 (4.54, 4.60) | 2.33 (2.30, 2.36) | 2.48 (2.45, 2.51) |
|  | 50% | 2.16 (2.13, 2.20) | 2.57 (2.54, 2.60) | 1.54 (1.51, 1.58) | 1.65 (1.61, 1.68) |
|  | 20% | 1.36 (1.32, 1.40) | 1.57 (1.53, 1.61) | 0.98 (0.94, 1.03) | 1.02 (0.98, 1.07) |
|  | 18% | 1.31 (1.27, 1.35) | 1.51 (1.47, 1.55) | 0.94 (0.89, 0.99) | 0.98 (0.93, 1.03) |
|  | 15% | 1.24 (1.20, 1.28) | 1.42 (1.37, 1.46) | 0.88 (0.83, 0.93) | 0.90 (0.93, 1.03) |
|  | 12% | 1.16 (1.12, 1.21) | 1.32 (1.28, 1.37) | 0.81 (0.76, 0.86) | 0.83 (0.77, 0.88) |
|  | 10% | 1.12 (1.07, 1.16) | 1.26 (1.22, 1.31) | 0.77 (0.71, 0.82) | 0.77 (0.72, 0.88) |
|  | 8% |  | 1.20 (1.16, 1.25) | 0.72 (0.67, 0.77) | 0.72 (0.66, 0.78) |
|  | 5% |  |  | 0.65 (0.59, 0.71) | 0.64 (0.57, 0.70) |
|  | 3% |  |  | 0.60 (0.54, 0.66) | 0.58 (0.51, 0.65) |
| **2021** | 100% | 3.42 (3.39, 3.44) | 4.13 (4.10, 4.16) | 2.46 (2.42, 2.49) | 2.73 (2.70, 2.76) |
|  | 50% | 2.00 (1.97, 2.03) | 2.37 (2.34, 2.40) | 1.54 (1.50, 1.58) | 1.72 (1.68, 1.76) |
|  | 20% | 1.29 (1.26, 1.33) | 1.49 (1.45, 1.53) | 0.95 (0.90, 0.99) | 1.04 (0.99, 1.09) |
|  | 18% | 1.25 (1.21, 1.29) | 1.43 (1.39, 1.47) | 0.91 (0.86, 0.95) | 0.99 (0.94, 1.04) |
|  | 15% | 1.18 (1.15, 1.22) | 1.35 (1.31, 1.39) | 0.84 (0.79, 0.89) | 0.92 (0.86, 0.97) |
|  | 12% | 1.12 (1.08, 1.16) | 1.27 (1.23, 1.31) | 0.78 (0.73, 0.83) | 0.84 (0.79, 0.89) |
|  | 10% |  | 1.22 (1.18, 1.26) | 0.73 (0.68, 0.79) | 0.79 (0.73, 0.84) |
|  | 8% |  |  | 0.69 (0.64, 0.74) | 0.74 (0.68, 0.79 |
|  | 5% |  |  | 0.62 (0.57, 0.68) | 0.65 (0.59, 0.72) |
|  | 3% |  |  | 0.58 (0.52, 0.64) | 0.60 (0.54, 0.66) |

* Living in a non-U.S. colony is used as the reference point for colonialism effect modification analyses

** PTB = Preterm birth, LBW = Low birthweight, GH = Gestational Hypertension, GD = Gestational Diabetes

*** Starting point for simulation “risk” was based on baseline (i.e., 2017) risk for outcome. PTB was 11.5%, LBW was 10.0%, GH was 4%, and GD was 3%.
